# Supplementary material for: Automated Microbial Library Generation Using the Bioinformatics Platform IDBac
Source: Molecules. 2022 Mar 22;27(7):2038. doi: 10.3390/molecules27072038 (PMC9000433; doi:10.3390/molecules27072038)
Supplement: Supplementary file 1 [file molecules-27-02038-s001.zip › molecules-1628128-supplementary.pdf]

molecules

# Automated Microbial Library Generation Using the Bioinformatics Platform IDBac

**Chase M. Clark [1], Linh Nguyen [2], Van Cuong Pham [2], Laura M. Sanchez [3] and Brian T. Murphy [1,*]**

[1] Department of Pharmaceutical Sciences, Center for Biomolecular Sciences, College of Pharmacy, University of Illinois at Chicago, 833 S. Wood St., Chicago, IL 60612, USA; cmclark8@wisc.edu
[2] Institute of Marine Biochemistry, Vietnam Academy of Science and Technology, Nghiado, Caugiay, Hanoi 10000, Vietnam; nguyenthuylinhorc@gmail.com (L.N.); phamvc@imbc.vast.vn (V.C.P.)
[3] Department of Chemistry and Biochemistry, University of California Santa Cruz, 1156 High Street, Santa Cruz, CA 95064, USA; lmsanche@ucsc.edu
* Correspondence: btmurphy@uic.edu; Tel.: +1-(312)-413-9057

| Ingredients | SWA | NZSG | A1 | M1 | ISP2 | SCA | PDA | ISP1 |
|---|---|---|---|---|---|---|---|---|
| Malt Extract (g) | 0 | 0 | 0 | 0 | 10 | 0 | 0 | 0 |
| Yeast Extract (g) | 0 | 5 | 4 | 0.4 | 4 | 0 | 0 | 3 |
| Soluble Starch (g) | 0 | 20 | 10 | 1 | 0 | 10 | 5 | 0 |
| Glucose (g) | 0 | 10 | 0 | 0 | 4 | 0 | 20 | 0 |
| 50% Glycerol (ml) | 0 | 0 | 0 | 0 | 0 | 0 | 0 | 0 |
| N-Z Amine A (g) | 0 | 5 | 0 | 0 | 0 | 0 | 0 | 0 |
| Casein (g) | 0 | 0 | 0 | 0 | 0 | 0.3 | 0 | 0 |
| Calcium Carbonate (g) | 0 | 3 | 0 | 0 | 0 | 0 | 0 | 0 |
| Casamino acids (g) | 0 | 0 | 0 | 0 | 0 | 0 | 0 | 0 |
| Casitone (g) | 0 | 0 | 0 | 0 | 0 | 0 | 0 | 5 |
| Potato extract (g) | 0 | 0 | 0 | 0 | 0 | 0 | 30 | 0 |
| Peptone (g) | 0 | 0 | 1 | 0.2 | 0 | 0 | 0 | 0 |
| Soytone (g) | 0 | 0 | 0 | 0 | 0 | 0 | 0 | 0 |
| Tryptone (g) | 0 | 0 | 0 | 0 | 0 | 0 | 0 | 0 |
| NaCl (g) | 0 | 0 | 0 | 0 | 0 | 0 | 0 | 0 |
| CaCO3 (g) | 0 | 3 | 0 | 0 | 0 | 0.02 | 0 | 0 |
| K2HPO4 (g) | 0 | 0 | 0 | 0 | 0 | 2 | 0 | 0 |
| KH2PO4 (g) | 0 | 0 | 0 | 0 | 0 | 0 | 0 | 0 |
| KNO3 (g) | 0 | 0 | 0 | 0 | 0 | 2 | 0 | 0 |
| MgSO4-7H20 (g) | 0 | 0 | 0 | 0 | 0 | 0.05 | 0 | 0 |
| 8 mg/mL FeSO4-7H2O (ml) | 0 | 0 | 0 | 0 | 0 | 0 | 0 | 0 |
| Agar (g) | 15 | 15 | 15 | 15 | 15 | 15 | 15 | 15 |
| Instant Ocean (g) | 30 | 30 | 30 | 30 | 30 | 30 | 30 | 30 |
| Filtered Lake Water (ml) | 1000 | 1000 | 1000 | 1000 | 1000 | 1000 | 1000 | 1000 |

**Table S1.** Media formulations used in this study.
